# Subarachnoid Hemorrhage Increases Level of Heme Oxygenase-1 and Biliverdin Reductase in the Choroid Plexus

**DOI:** 10.3389/fncel.2020.593305

**Published:** 2020-11-26

**Authors:** Peter Solár, Václav Brázda, Shahaf Levin, Alemeh Zamani, Radim Jančálek, Petr Dubový, Marek Joukal

**Affiliations:** ^1^Department of Anatomy, Faculty of Medicine, Cellular and Molecular Neurobiology Research Group, Masaryk University, Brno, Czechia; ^2^Department of Neurosurgery – St. Anne’s University Hospital Brno, Faculty of Medicine, Masaryk University, Brno, Czechia; ^3^Department of Neurosurgery, St. Anne’s University Hospital Brno, Brno, Czechia; ^4^Institute of Biophysics of the Czech Academy of Sciences, Brno, Czechia

**Keywords:** subarachnoid hemorrhage, choroid plexus, heme oxygenase-1, biliverdin reductase, macrophages

## Abstract

Subarachnoid hemorrhage is a specific, life-threatening form of hemorrhagic stroke linked to high morbidity and mortality. It has been found that the choroid plexus of the brain ventricles forming the blood-cerebrospinal fluid barrier plays an important role in subarachnoid hemorrhage pathophysiology. Heme oxygenase-1 and biliverdin reductase are two of the key enzymes of the hemoglobin degradation cascade. Therefore, the aim of present study was to investigate changes in protein levels of heme oxygenase-1 and biliverdin reductase in the rat choroid plexus after experimental subarachnoid hemorrhage induced by injection of non-heparinized autologous blood to the cisterna magna. Artificial cerebrospinal fluid of the same volume as autologous blood was injected to mimic increased intracranial pressure in control rats. Immunohistochemical and Western blot analyses were used to monitor changes in the of heme oxygenase-1 and biliverdin reductase levels in the rat choroid plexus after induction of subarachnoid hemorrhage or artificial cerebrospinal fluid application for 1, 3, and 7 days. We found increased levels of heme oxygenase-1 and biliverdin reductase protein in the choroid plexus over the entire period following subarachnoid hemorrhage induction. The level of heme oxygenase-1 was the highest early (1 and 3 days) after subarachnoid hemorrhage indicating its importance in hemoglobin degradation. Increased levels of heme oxygenase-1 were also observed in the choroid plexus epithelial cells at all time points after application of artificial cerebrospinal fluid. Biliverdin reductase protein was detected mainly in the choroid plexus epithelial cells, with levels gradually increasing during subarachnoid hemorrhage. Our results suggest that heme oxygenase-1 and biliverdin reductase are involved not only in hemoglobin degradation but probably also in protecting choroid plexus epithelial cells and the blood-cerebrospinal fluid barrier from the negative effects of subarachnoid hemorrhage.

## Introduction

Subarachnoid hemorrhage is a specific form of hemorrhagic stroke with incidence rates of 0.5–28 cases worldwide per 100,000 people annually, although regional rates vary widely (Etminan et al., 2019). Despite this rate, the prognosis for subarachnoid hemorrhage remains poor, with a third of the patients dying and a further third surviving in a dependent state (Yeo et al., 2019). Aneurysm rupture causes a rapid increase in intracranial pressure resulting in compression of brain tissue in the first few seconds (Sehba et al., 2011). In addition, there is a correlation between the extent of intracranial pressure rise and the early-onset of brain injury as well as mortality (Zoerle et al., 2015).

The choroid plexus of the brain ventricles is composed of cuboidal epithelial cells that adhere to a highly vascularized stroma containing fenestrated capillaries, loose connective tissue and immune cells. The main function of the choroid plexus is the production of almost 60–80% of cerebrospinal fluid and acting as the interface between blood and the cerebrospinal fluid – the so-called blood-cerebrospinal fluid barrier. The permeability of the blood-cerebrospinal fluid barrier is mainly regulated by the tight junctions between adjacent cuboidal epithelial cells. The epiplexus Kolmer cells adhering to the ventricular side of the cuboidal epithelial cells are considered epiplexus macrophages (Hosoya and Fujita, 1973; Ling et al., 1998; McMenamin et al., 2003; Wolburg and Paulus, 2010). In a recent paper, we described an increase in the number of epiplexus macrophages as a consequence of subarachnoid hemorrhage as well as increased intracranial pressure induced by applying artificial cerebrospinal fluid (Solár et al., 2020a).

Following subarachnoid hemorrhage, red blood cells are lysed in the subarachnoid space and release cell-free hemoglobin (Sadrzadeh et al., 1987; Regan and Panter, 1993; Hugelshofer et al., 2019; Buehler et al., 2020). It was found that cell-free hemoglobin has many harmful properties including the initiation of inflammation, free radical reactions as well as vasoconstriction caused by nitric oxide depletion (Gram et al., 2013). The toxicity of cell-free hemoglobin is potentiated by its ability to move from cerebrospinal fluid to brain parenchyma, cerebral arteries, and arterioles resulting in microvascular thrombosis, oxidative stress damage, cortical spreading depolarization, and cerebral vasospasm (Hugelshofer et al., 2019).

Heme oxygenase-1 and biliverdin reductase are the main enzymes involved in hemoglobin degradation (Shibahara et al., 2002). There are two major types of heme oxygenase; heme oxygenase-1 is the inducible isoform while heme oxygenase-2 is constitutively expressed in various tissues including the brain, testes and endothelia and mediates hemoglobin oxidation to biliverdin (Willis et al., 1996). Schallner et al. (2015) suggested that heme oxygenase-1 is the major enzyme responsible for clearing heme from the subarachnoid space following subarachnoid hemorrhage. Biliverdin is reduced to bilirubin by biliverdin reductase, which has been found also in brain tissue, where it appears to be co-expressed with heme oxygenase isoforms (Ewing et al., 1993; Maines, 2005). We hypothesize that these two enzymes may contribute to the development of the inflammatory response in the choroid plexus following subarachnoid hemorrhage described in our previous work (Solár et al., 2020a). Therefore, the aim of our present experiments was to monitor the time-course of heme oxygenase-1 and biliverdin reductase protein levels in the choroid plexus following subarachnoid hemorrhage or application of artificial cerebrospinal fluid as control. The monitoring was performed using immunohistochemical detection combined with image analysis and Western blot analysis.

## Materials and Methods

### Animals and Surgical Procedure

In this study we used 94 adult male rats (Wistar 200–250 gr; Animal Breeding Facility, Masaryk University, Czechia). All experimental procedures were performed aseptically and in accordance with the protocols approved by the Ethical Committee of Masaryk University, Brno, and the Departmental Committee of the Ministry of Education, Youth and Sports, Czechia (Approval No.: MŠMT 21101/2016-3). A mixture of 5% ketamine (100 mg/kg) and 2% xylazine (10 mg/kg) was intraperitoneally administrated to anesthetize the rats.

Subarachnoid hemorrhage was induced following the standardized method originally published by Solomon et al. and modified by other authors, including our group (Solomon et al., 1985; d’Avella et al., 1996; Matz et al., 1996; Solár et al., 2020a). In brief, cannulation of the caudal artery was performed in the subarachnoid hemorrhage group. To reach the atlas, occipital bone and suboccipital membrane, a midline suboccipital incision was made. Then, the animal was placed in a stereotaxic apparatus (Kopf Instruments, Tujunga, CA, United States) and a syringe with a 30G needle was placed on the manipulating arm of the stereotaxic apparatus tilted at 60 degrees from the horizontal plane. Under high magnification in a surgical microscope, 200 μl of non-heparinized autologous arterial blood was injected into the cisterna magna within 60 s (d’Avella et al., 1996).

The animals in the artificial cerebrospinal fluid group were also injected within 60 s, but received 200 μl of artificial cerebrospinal fluid consisting of 130 mM NaCl, 3.0 mM KCl, 1.2 mM NaH_2_PO_4_, 20 mM NaHCO_3_, 1.3 mM MgCl_2_, 2.4 mM CaCl_2_, and 10 mM Glucose (Hylden and Wilcox, 1980). The artificial cerebrospinal fluid for each experiment was prepared fresh and the pH was set to 7.40 ± 0.05. To prevent leakage of blood or artificial cerebrospinal fluid from the cisterna magna, the needle was slowly withdrawn 2 min after injection, and the membrane puncture was closed with a gelatin sponge. The muscles and the skin were closed using 4-0 silk suture.

### Immunohistochemical Staining

The rats of subarachnoid hemorrhage and artificial cerebrospinal fluid group were left to survive for 1 (*n* = 10 subarachnoid hemorrhage; *n* = 10 artificial cerebrospinal fluid), 3 (*n* = 10 subarachnoid hemorrhage; *n* = 10 artificial cerebrospinal fluid), and 7 days (*n* = 10 subarachnoid hemorrhage; *n* = 10 artificial cerebrospinal fluid) and along with a naïve group (*n* = 6) were sacrificed by CO2 inhalation, then 500 ml heparinized (1,000 units/500 ml) phosphate–buffered saline (PBS; pH 7.4) was used for transcardial perfusion. This was followed by 500 ml of Zamboni’s fixative (Zamboni and DeMartino, 1967).

The brains were removed immediately and inspected for successful injection scored as the presence of blood in the subarachnoid cisterns and the basal surface of the brain in the subarachnoid hemorrhage group, and no blood in the subarachnoid space in the artificial cerebrospinal fluid group. The brains were kept in Zamboni’s fixative for 72 h, then rinsed in 10% sucrose and embedded in Tissue-Tek OCT compound (Miles; Elkhart, IN, United States). Serial coronal cryostat sections (20 μm) were prepared (Leica 1800 cryostat; Leica Microsystem, Wetzlar, Germany) and mounted onto chrome-alum covered microscopic slides.

Heme oxygenase-1 and biliverdin reductase proteins were detected simultaneously in brain section arrays of naïve, subarachnoid hemorrhage, and artificial cerebrospinal fluid animals using indirect immunohistochemical staining under the same conditions. In brief, the sections were rinsed with PBS containing 0.3% bovine serum albumin and 0.1% Tween-20, treated with 3% normal donkey serum for 30 min, and incubated overnight with a rabbit polyclonal antibody against heme oxygenase-1 (1:200; ab13242, Abcam, Burlingame, CA, United States) or BVR-A (1:100; PA1-26089, Thermo Fisher Scientific, Waltham, MA, United States) in a humid chamber at room temperature (21–23°C). Cy5-conjugated donkey anti-rabbit secondary antibody (Jackson, 1:100) was applied at room temperature for 90 min to visualize the immune reactions. Control sections were incubated in parallel, without the primary antibody.

Immunofluorescence images were acquired and analyzed using a Nikon Eclipse NI-E epifluorescence microscope equipped with a stabilized power supply for the lamp housing and a Nikon DS-Ri1 camera (Nikon, Prague, Czechia).

### Double Immunohistochemical Staining

Double immunostaining of heme oxygenase-1 and ED1 or ED2 was performed to examine the expression of heme oxygenase-1 in the choroid plexus macrophages. The ED1 (anti-CD68) and ED2 (anti-CD163) antibodies are used to detect macrophages with activated phagocytosis and resident tissue macrophages, respectively (Dijkstra and Damoiseaux, 1993). Sections were incubated with rabbit polyclonal anti-heme oxygenase-1 antibody and treated with AlexaFluor 488-conjugated goat anti-rabbit secondary antibody (Jackson, 1:50; West Grove, PA, United States) for 90 min. After thorough washing, a primary mouse monoclonal antibody against ED1 (1:100; MCA341R, Serotec, Hercules, CA, United States) or ED2 (1:200; MCA342R, Serotec, Hercules, CA, United States) was applied at room temperature for 240 min (ED1) or 16 h (ED2) followed by Cy5-conjugated donkey anti-mouse secondary antibodies (Jackson, 1:100) applied at room temperature for 90 min. Sections were mounted and analyzed in the colocalization module of NIS Elements software (Nikon, Czechia).

### Image Analysis

Digital images (magnification 200×) were acquired from at least 10 sections selected at 60 μm intervals from serial brain sections containing the choroid plexus of lateral ventricles. All images of the choroid plexus for each group of animals were acquired with identical settings for the camera, optics and the lamp as well as the same exposure conditions; the acquired images were stored in the TIFF format. The analyses were carried out according to our previously published protocols (Dubový et al., 2002, 2013) using the NIS-Elements AR Analysis software (version 4.20.00, Nikon, Prague, Czechia) by an investigator who was blind to the animal groups.

To measure the intensity of heme oxygenase-1 and biliverdin reductase immunofluorescence in clearly limited choroid plexus epithelial cells, a binary mask was created by manually detecting cell boundaries and excluding nuclei. Immunofluorescence intensities of at least 100 epithelial cells for each animal group were measured after subtraction of background. The intensity of heme oxygenase-1 and biliverdin reductase immunofluorescence were expressed as means ± SD. To quantify the number of heme oxygenase-1 positive Kolmer cells, the choroid plexus area was first manually determined in the images and the extent measured, edited when needed, and heme oxygenase-1 positivity in the Kolmer cells was correlated with the positions of cell nuclei stained by Hoechst and counted manually in the defined CP area. The number of the heme oxygenase-1 positive Kolmer cells per 1 mm^2^ of the choroid plexus area was expressed as means ± SD.

### Western Blot Analysis

We used Western blot analysis to validate the semiquantitative changes of heme oxygenase-1 and biliverdin reductase levels in rat choroid plexus obtained by measuring immunofluorescence intensity. The rats of naïve group (*n* = 4), subarachnoid hemorrhage as well as artificial cerebrospinal fluid group were left to survive for 1 (*n* = 4 subarachnoid hemorrhage; *n* = 4 artificial cerebrospinal fluid), 3 (*n* = 4 subarachnoid hemorrhage; *n* = 4 artificial cerebrospinal fluid), and 7 days (*n* = 4 subarachnoid hemorrhage; *n* = 4 artificial cerebrospinal fluid) and sacrificed by CO2 inhalation. Under aseptic conditions, the choroid plexus of both brain hemispheres were removed, immersed in protease inhibitor and phosphatase inhibitor cocktails (Roche, Germany), and flash-frozen in liquid nitrogen, and stored at −80°C until analysis. Tissue samples of each group of animals were collected and homogenized in PBS with 0.1% Triton X-100 and protease inhibitors (LaRoche, Switzerland), and centrifuged at 10,000 × *g* for 5 min at 4°C. Proteins were further separated by SDS – polyacrylamide gel electrophoresis as previously published (Brazda et al., 2006; Dubový et al., 2013). Blots were blocked with 1% BSA in PBST (3.2 mM Na2HPO4, 0.5 mM KH2PO4, 1.3 mM KCl, 135 mM NaCl, 0.05% Tween 20, and pH 7.4) for 1 h at room temperature and incubated with β-actin rabbit polyclonal antibody (1:1000; Cell Signaling Technology; Netherlands), anti-heme oxygenase-1 rabbit polyclonal antibody (1:250; ab13242, Abcam, Burlingame, CA, United States) or anti-biliverdin reductase rabbit polyclonal antibody (1:250; PA1-26089, Thermo Fisher Scientific, Waltham, MA, United States) overnight. The antibodies each recognize a single band at 32 and 36 kDa corresponding to heme oxygenase-1 and biliverdin reductase proteins, respectively. These protein bands were also found using the same or similar antibodies applied for Western blot analysis of heme oxygenase-1 and biliverdin reductase protein in other types of tissues (Jansen et al., 2017; Xu et al., 2018; Sharma et al., 2019; Stec et al., 2020). Blots were further washed in PBST and incubated with peroxidase-conjugated anti-rabbit or anti-mouse IgG (Sigma, 1:1000) at room temperature for 1 h. The ECL detection kit (Amersham) was used to visualize protein bands on the chemiluminometer reader LAS-3000 and analyzed using densitometry image software. The intensity of the bands was measured in triplicate blots and is expressed as mean ± SD.

### Statistical Analysis

The data of immunoquantification and Western blot analysis in naïve, artificial cerebrospinal fluid and subarachnoid hemorrhage groups were compared using multiple comparisons of mean ranks for all groups (Kruskal–Wallis with Bonferroni *post hoc* test; *p* < 0.05; *p* < 0.01) in STATISTICA 13.2 software (StatSoft, Tulsa, OK, United States).

## Results

All experimental animals that underwent application of intrathecal blood (subarachnoid hemorrhage group) or artificial cerebrospinal fluid survived the experiments. The application was successful in all animals as indicated by the presence of blood in the subarachnoid space in the (subarachnoid hemorrhage group) or no blood in the artificial cerebrospinal fluid group. All animals were included in the analyses of heme oxygenase-1 and biliverdin reductase in the choroid plexus.

### Levels of Heme Oxygenase-1 Protein Following Subarachnoid Hemorrhage or Application of Artificial Cerebrospinal Fluid

**FIGURE 1:**
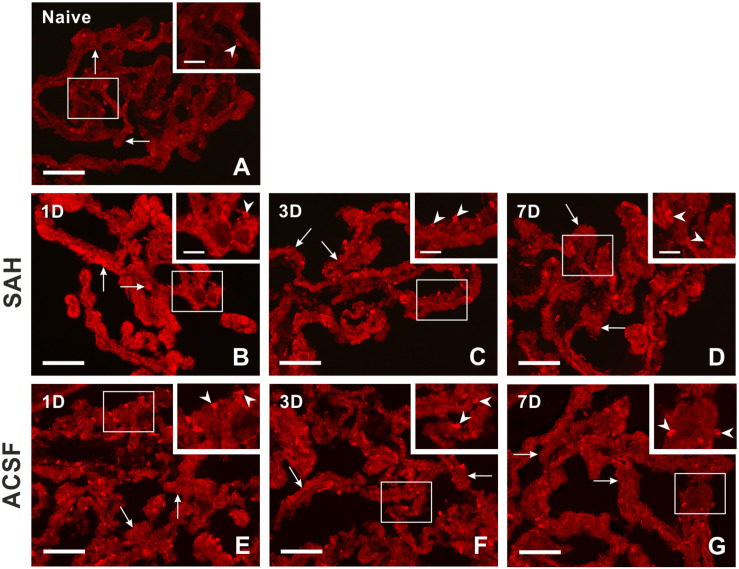
Representative pictures showing heme oxygenase-1 immunostaining in the choroid plexus from naïve rats, 1, 3, and 7 days (1D, 3D, and 7D) after induction of subarachnoid hemorrhage (SAH) as well as artificial cerebrospinal fluid (ACSF) application. Arrows indicate heme oxygenase-1 positive epithelial cells and arrowheads heme oxygenase-1 positive Kolmer cells in naïve rats **(A)**, 1 **(B)**, 3 **(C)**, and 7 days **(D)** after induction of subarachnoid hemorrhage and 1 **(E)**, 3 **(F)**, and 7 days **(G)** after artificial cerebrospinal fluid application. Insets at top right illustrate higher magnifications of the regions indicated by the box in the main picture. Scale bars – main images 80 μm; insets 20 μm.

**FIGURE 2:**
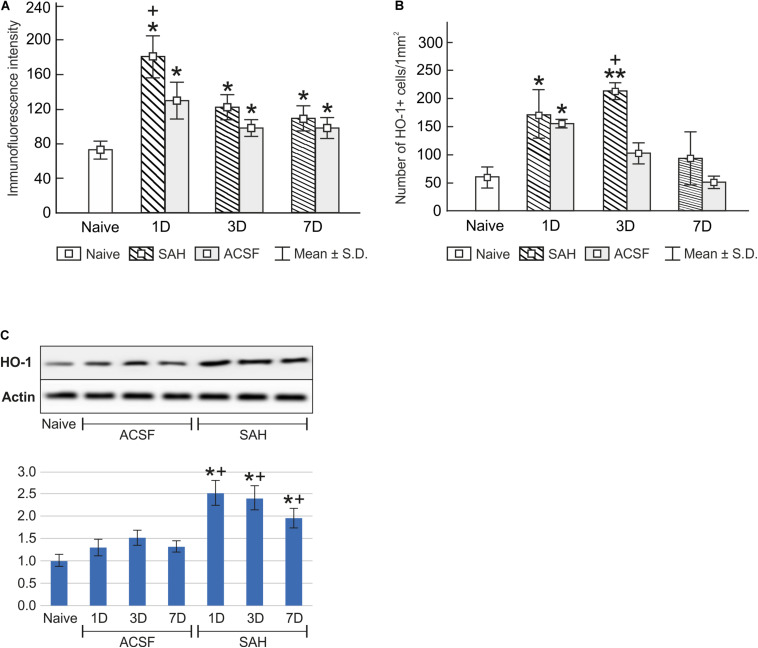
Immunoquantification of heme oxygenase-1 in the choroid plexus of naïve, subarachnoid hemorrhage (SAH), and artificial cerebrospinal fluid (ACSF) rats at 1, 3, and 7 days after the operation. Graphs show mean intensity (±SD) of heme oxygenase-1 immunofluorescence in choroid plexus epithelial cells **(A)** and numbers of heme oxygenase-1 positive (HO-1+) Kolmer cells per mm^2^ **(B)**. Representative Western blot analysis comparing the levels of heme oxygenase-1 (HO-1) in the choroid plexus in different experimental groups is shown in the upper panels in panel **(C)**. Actin demonstrates equal protein loading. Densitometry of heme oxygenase-1 protein bands after normalization to actin is shown in the lower panels in panel **(C)**. The intensities of heme oxygenase-1 bands from naïve choroid plexus were taken as 1. *Significant difference compared to the choroid plexus from naïve rats (*p < 0.05; **p < 0.01). +Significant difference compared to the choroid plexus from artificial cerebrospinal fluid rats (+*p* < 0.05).

Immunostaining for heme oxygenase-1 was found in both cuboidal epithelial cells and epiplexus Kolmer cells of the choroid plexus of naïve, subarachnoid hemorrhage, and artificial cerebrospinal fluid animals (Figure 1A–G). Immunohistochemical staining showed a significantly increased intensity of heme oxygenase-1 immunofluorescence in the choroid plexus epithelial cells 1, 3, and 7 days (*p* < 0.05) following subarachnoid hemorrhage induction or artificial cerebrospinal fluid application compared to naïve animals with the highest intensity at one-day of subarachnoid hemorrhage. The intensity of heme oxygenase-1 immunofluorescence was significantly increased one-day (*p* < 0.05) after induction of subarachnoid hemorrhage when compared with artificial cerebrospinal fluid animals at the same time point (Figure 2A). The number of heme oxygenase-1 positive Kolmer cells was significantly increased in the choroid plexus of animals 1 (*n* = 166.3 ± 40/mm^2^; *p* < 0.05) and 3 days (*n* = 203.1 ± 18.5/mm^2^; *p* < 0.01) following subarachnoid hemorrhage induction when compared to naïve animals (*n* = 64 ± 25.2/mm^2^). Moreover, immunostaining also showed a significant increased number of heme oxygenase-1 positive cells in the choroid plexus of animals one-day (*n* = 178.4 ± 45.12/mm^2^; *p* < 0.05) after artificial cerebrospinal fluid application compared to naïve rats (*n* = 63.69 ± 25.2/mm^2^). The number of heme oxygenase-1 positive Kolmer cells was significantly higher at 3 days (*p* < 0.05) after subarachnoid hemorrhage induction when compared to artificial cerebrospinal fluid group of animals at the same time point (Figure 2B). No erythrophagocytosis was detected in heme oxygenase-1 positive Kolmer cells.

Changes in heme oxygenase-1 levels in the choroid plexus of subarachnoid hemorrhage and artificial cerebrospinal fluid animals were confirmed by Western blot analysis. Levels of heme oxygenase-1 protein were significantly increased in the choroid plexus removed at 1 (2.5-fold; *p* < 0.05), 3 (2.4-fold; *p* < 0.05), and 7 days (1.9-fold; *p* < 0.05) after subarachnoid hemorrhage induction. A distinct increase was also found 1 (1.3-fold), 3 (1.5-fold), and 7 days (1.3-fold) following artificial cerebrospinal fluid application compared to the choroid plexus from naïve animals (Figure 2C and Supplementary Figures 1, 3), although this increase was not significant (*p* = 0.243).

**FIGURE 3:**
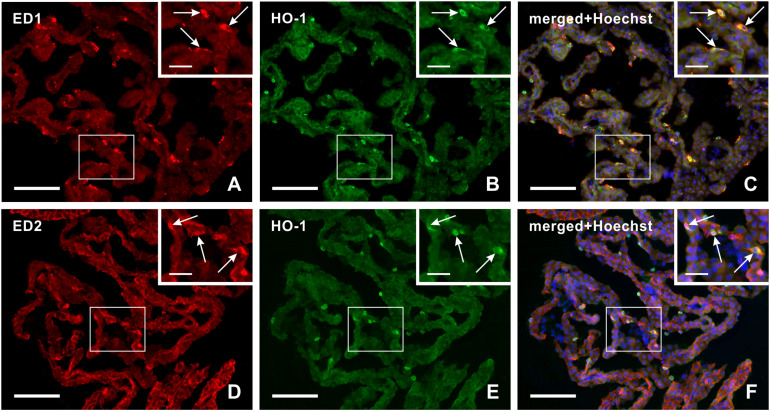
Representative pictures illustrating double immunostaining to detect heme oxygenase-1 (HO-1) positive ED1+ **(A–C)** or ED2+ **(D–F)** macrophages in the choroid plexus 3 days after subarachnoid hemorrhage. Merged pictures with Hoechst stained nuclei **(C,F)** proved that heme oxygenase-1 positive Kolmer cells are either activated (ED1+) or resident (ED2+) macrophages (arrows). Scale bars – main images 80 μm; insets 20 μm.

Double immunohistochemical staining demonstrated that heme oxygenase-1 positive cells in the epiplexus position from subarachnoid hemorrhage animals displayed positivity for both activated (ED1+) and resident (ED2+) macrophages (Figure 3A–F).

### Levels of Biliverdin Reductase Protein Following Subarachnoid Hemorrhage or Artificial Cerebrospinal Fluid Application

**FIGURE 4:**
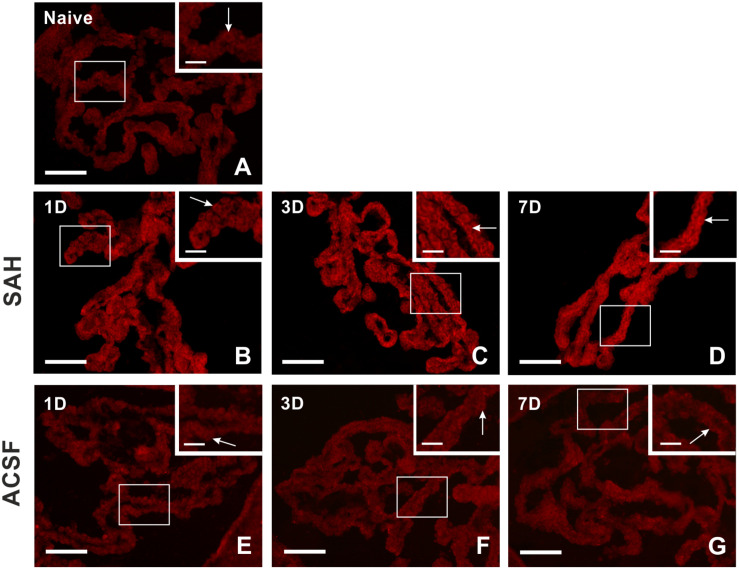
Representative pictures showing biliverdin reductase immunostaining in the choroid plexus from naïve rats, 1, 3, and 7 days (1D, 3D, and 7D) after induction of subarachnoid hemorrhage (SAH) as well as artificial cerebrospinal fluid (ACSF) application. Arrows indicate biliverdin reductase positive epithelial cells in the naïve rats **(A)**, 1 **(B)**, 3 **(C)**, and 7 days **(D)** after induction of subarachnoid hemorrhage and 1 **(E)**, 3 **(F)**, and 7 days **(G)** after artificial cerebrospinal fluid application. Insets at top right illustrate higher magnifications of the regions indicated by the box in the main picture. Scale bars – main images 80 μm; insets 20 μm.

**FIGURE 5:**
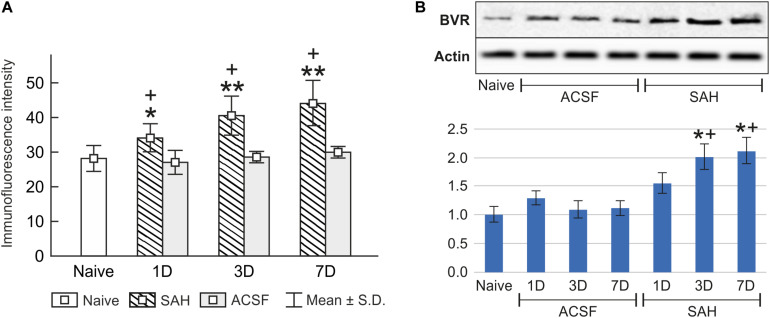
Immunoquantification of biliverdin reductase in the choroid plexus of naïve, subarachnoid hemorrhage (SAH), and artificial cerebrospinal fluid (ACSF) rats at 1, 3, and 7 days after the operation. The mean intensity (±SD) of biliverdin reductase immunofluorescence in choroid plexus epithelial cells is shown in panel **(A)** and Western blot analysis in panel **(B)**. Representative blots comparing biliverdin- reductase (BVR) in the choroid plexus from different animal groups is shown in the upper panels. Actin demonstrates equal protein loading. Densitometry of biliverdin reductase protein bands after normalization to actin is shown in the lower panels. The intensities of biliverdin reductase-1 bands from naïve CP were taken as 1. *Significant difference compared to the choroid plexus from naïve rats (*p < 0.05; **p < 0.01). +Significant difference compared to the choroid plexus from artificial cerebrospinal fluid rats (+*p* < 0.05).

Immunohistochemical staining showed biliverdin reductase immunopositivity only in the cuboidal epithelial cells of the choroid plexus in all experimental groups (Figure 4). The intensity of biliverdin redutase immunofluorescence in the choroid plexus was significantly increased 1 (*p* < 0.05), 3 (*p* < 0.01), and 7 days (*p* < 0.01) after subarachnoid hemorrhage induction when compared to naïve animals. Intensity of biliverdin reductase immunofluorescence gradually increased over time following subarachnoid hemorrhage, but the increase was significant 3 and 7 days after subarachnoid hemorrhage when compared to artificial cerebrospinal fluid animals (*p* < 0.05) at the same time points (Figure 5A).

The changes in biliverdin reductase levels in the choroid plexus of subarachnoid hemorrhage and artificial cerebrospinal fluid animals were confirmed by Western blot analysis. Significantly increased biliverdin reductase protein levels were found 3 (twofold; *p* < 0.05), and 7 days (twofold; *p* < 0.05) following subarachnoid hemorrhage when compared to artificial cerebrospinal fluid group of animals or naïve rats (Figure 5B and Supplementary Figures 2, 3).

## Discussion

### Subarachnoid Hemorrhage Increases Levels of Heme Oxygenase-1 in the Choroid Plexus Cells

Increased heme oxygenase-1 immunostaining was induced at the place of the cerebral cisterns suggesting that subarachnoid hemoglobin concentration plays an important role in regulating heme oxygenase-1 levels (Matz et al., 1996) and that it is the major enzyme responsible for clearing blood from the subarachnoid space following subarachnoid hemorrhage (Schallner et al., 2015). In addition, heme oxygenase-1 found in neurons, microglia and astrocytes may alleviate hemorrhage-induced stress injury and reduce brain damage after subarachnoid hemorrhage (Turner et al., 1999; Nakaso et al., 2000). It was suggested that increased heme oxygenase-1 observed in choroid plexus cells after experimental intraventricular hemorrhage is probably induced in response to the presence of blood in the cerebrospinal fluid (Gram et al., 2014). Degradation of erythrocytes in the cerebrospinal fluid leads to the deposition of hemoglobin, which is bound by the high-affinity binding protein haptoglobin (Lucke-Wold et al., 2016; Blackburn et al., 2018). The haptoglobin-hemoglobin complex is recognized by the scavenger receptor CD163 that has been also described as an ED2 antigen constitutively expressed in a subpopulation of mature tissue macrophages (Fabriek et al., 2005; Polfliet et al., 2006). These macrophages are able to phagocyte red blood cells apart from being able to scavenge and endocytose haptoglobin-hemoglobin complexes from the cerebrospinal fluid (Galea et al., 2012). As heme is released from hemoglobin, heme oxygenase-1 degrades it to Fe^2+^, biliverdin, and carbon monoxide inside the endosomes (Garton et al., 2017b).

Our results of immunohistochemical analysis confirmed by Western blot assessment demonstrated increased levels of total heme oxygenase-1 protein in the choroid plexus following induction of subarachnoid hemorrhage. Using heme oxygenase-1 immunostaining, we detected its highest immunopositivity in both choroid plexus epithelial cells and Kolmer cells early after subarachnoid hemorrhage (1 and 3 days), supporting a role for heme oxygenase-1 in hemoglobin degradation. In addition, double immunostaining revealed that heme oxygenase-1 positive Kolmer cells displayed positivity for both activated (ED1+) and resident (ED2+) macrophages. This suggests that hemoglobin detoxification from the subarachnoid space after subarachnoid hemorrhage is not carried out only by resident macrophages (ED2+) and their CD163 mediated haptoglobin-hemoglobin uptake, but also via phagocytosis by activated (ED1+) macrophages.

Epiplexus macrophages are in direct contact with blood degradation products in the cerebrospinal fluid after subarachnoid hemorrhage. Hematin granules and breakdown products of erythrocytes were found in the epiplexus macrophages after induction of subarachnoid hemorrhage (Liszczak et al., 1984). Our previous study demonstrated increased numbers of Kolmer cells with ED1 and ED2 immunopositivity over the duration of subarachnoid hemorrhage (Solár et al., 2020a). We show here that both ED1 and ED2 immunopositive Kolmer cells also display heme oxygenase-1 immunostaining. Based on these results, we can assume that these epiplexus macrophages may significantly contribute to the phagocytosis and clearance of blood degradation products in the cerebrospinal fluid after subarachnoid hemorrhage.

Blood degradation products in the cerebrospinal fluid after subarachnoid hemorrhage also lead to inflammation in the choroid plexus (Kwon et al., 2015; Garton et al., 2017a; Solár et al., 2020b) leading to alterations in tight junction proteins with consequent disruption of the blood-cerebrospinal fluid barrier and cerebrospinal fluid hypersecretion (Simard et al., 2011; Karimy et al., 2017). It is known that overexpression of heme oxygenase-1 in epithelial cells improves epithelial barrier function by increasing the expression of the tight junction protein occludin (Zhang et al., 2017). Therefore, the increased level of heme oxygenase-1 in choroid plexus epithelial cells after subarachnoid hemorrhage we found in our experimental animals may contribute to maintaining blood-cerebrospinal fluid barrier integrity. Moreover, it was assumed that heme oxygenase-1 upregulation may have powerful antioxidant, anti-apoptotic and anti-inflammatory effects, mainly through the parallel activation of other enzymes such as biliverdin reductase that generates bilirubin and CO which probably has vasodilatory and cytoprotective effects (Morita et al., 1995; Suzuki et al., 1999; Almeida et al., 2012; Nitti et al., 2018).

### Increased Level of Biliverdin Reductase Is Induced in the Choroid Plexus Cells Following Subarachnoid Hemorrhage

Biliverdin, an oxidative product of heme oxygenase-mediated heme degradation, is subsequently reduced to bilirubin by biliverdin reductase (McDonagh, 2001), a soluble enzyme that can use NADH or NADPH as the electron donor (Noguchi et al., 1979; Kikuchi et al., 2001). Biliverdin reductase expression is constant in all tissues under basal conditions and is upregulated by its substrate biliverdin (Wegiel and Otterbein, 2012). Biliverdin reductase has been found in brain tissue, where it appears to be co-expressed with heme oxygenase isoforms (Ewing et al., 1993; Maines, 2005).

We observed significantly increased intensity of biliverdin reductase immunofluorescence in choroid plexus epithelial cells 1, 3, and 7 days following subarachnoid hemorrhage induction. In addition, this biliverdin reductase immunostaining gradually increased over the entire duration of subarachnoid hemorrhage which was confirmed by Western blot analysis. The results of increased immunostaining for heme oxygenase-1 and biliverdin reductase in choroid plexus epithelial cells may indicate their involvement in heme degradation early after experimental subarachnoid hemorrhage (1 and 3 days).

Biliverdin reductase enzymatically generates bilirubin, which is highly lipophilic and protects against lipid peroxidation (Sedlak et al., 2009). Bilirubin reacts with oxyradicals and generates biliverdin via oxidative conversion, and that is immediately reduced back to bilirubin through biliverdin reductase dependent catalysis (Baranano et al., 2002; Chang et al., 2005; McDonagh, 2010).

The protective antioxidative effect of bilirubin produced by biliverdin reductase (Jansen and Daiber, 2012; Mendez et al., 2013; Chen et al., 2018) is concentration-dependent (Kapitulnik, 2004; Basiglio et al., 2010). It was shown that the concentration of bilirubin is increased during the first week following subarachnoid hemorrhage (Vermeulen et al., 1989) and its high concentration in the cerebrospinal fluid was associated with a lower risk of cerebral vasospasm 5–7 days from subarachnoid hemorrhage induction (Suzuki et al., 2003). Moreover, it was found that biliverdin reductase facilitates hematoma resolution and attenuates inflammation following hemorrhagic stroke (Zhang et al., 2018). Taken together, the results of increased biliverdin reductase levels found in the choroid plexus of experimental rats also at day 7 may be associated with the antioxidant effect of biliverdin reductase activated in the later phases following subarachnoid hemorrhage.

### Increased Intracranial Pressure Induces a Higher Level of Heme Oxygenase-1 in Choroid Plexus Cells

Increased intracranial pressure due to subarachnoid hemorrhage was mimicked by the application of artificial cerebrospinal fluid into the cisterna magna in artificial cerebrospinal fluid group of animals (Solomon et al., 1985; Prunell et al., 2003). The volume of blood or artificial cerebrospinal fluid injected into the cistern magna was equal for all animals in both subarachnoid hemorrhage and artificial cerebrospinal fluid groups. Application of artificial cerebrospinal fluid is commonly used as a control in experimental models of inflammatory diseases as well as for intrathecal drug application with no evidence of immunogenicity (Hylden and Wilcox, 1980; Hernangómez et al., 2016). A previous study found that application of blood into the cisterna magna induces motor impairment not found in animals receiving artificial cerebrospinal fluid (Germanò et al., 1994).

It was found that injection of 200 μl of artificial cerebrospinal fluid into the cisterna magna leads to a sudden increase in intracranial pressure from basal values to values exceeding 100 mmHg within 15 s that subsequently gradually decreases to near-normal values within 2.5 min (Prunell et al., 2002). Injection speed is crucial in intracranial pressure dynamics. Rapid application (artificial cerebrospinal fluid or blood) within 60 s leads to higher intracranial pressure compared to slower injection speeds (10, 30, or 60 min). A sudden increase in intracranial pressure due to rapid application may result in harmful changes, including impairment of cerebral autoregulation as well as neuronal cell loss (Conzen et al., 2019).

In our study, we found increased levels of heme oxygenase-1 and biliverdin reductase in choroid plexus cells after induction of subarachnoid hemorrhage, but increased heme oxygenase-1 was also detected on the first day after artificial cerebrospinal fluid application. This increased heme oxygenase-1 level can be considered a defense mechanism of choroid plexus cells against oxidative stress triggered by intracranial hypertension (Wd et al., 1999; Conzen et al., 2019). On the other hand, biliverdin reductase level was not significantly altered in the choroid plexus of animals after artificial cerebrospinal fluid administration suggesting that biliverdin reductase is not affected by intracranial hypertension-driven oxidative stress.

## Conclusion

In conclusion, we found increased levels of heme oxygenase-1 and biliverdin reductase proteins in the choroid plexus over the entire period following subarachnoid hemorrhage induction. The level of heme oxygenase-1 was the highest early after subarachnoid hemorrhage indicating its importance in hemoglobin degradation. Interestingly, increased heme oxygenase-1 was also detected in the choroid plexus after artificial cerebrospinal fluid application for one-day, indicating that the sudden elevation of intracranial pressure after subarachnoid hemorrhage may also contribute to alteration of heme oxygenase-1 levels. Heme oxygenase-1 positivity was found in both CP epithelial cells as well as ED1+ and ED2+ epiplexus macrophages.

Biliverdin reductase protein was detected mainly in the choroid plexus epithelial cells, with levels gradually increasing during subarachnoid hemorrhage. In contrast to biliverdin reductase, heme oxygenase-1 expression shifted from epiplexus macrophages in the early stage, to choroid plexus epithelial cells in the later stage following subarachnoid hemorrhage induction. This finding suggests a beneficial role for both heme oxygenase-1 and biliverdin reductase not only in hemoglobin degradation but also in cytoprotection of choroid plexus epithelial cells and the blood-cerebrospinal fluid barrier after subarachnoid hemorrhage.

## Data Availability Statement

The raw data supporting the conclusions of this article will be made available by the authors, without undue reservation.

## Ethics Statement

The animal study was reviewed and approved by Ethical Committee of Masaryk University, Brno.

## Author Contributions

PS and MJ designed the research, performed the experiments, and wrote the manuscript. VB performed western blot analysis, analyzed the data, and wrote the manuscript. SL and AZ prepared and analyzed the samples for immunohistochemistry and wrote the manuscript. PD and RJ analyzed the data and wrote the manuscript. All authors contributed to the article and approved the submitted version.

## Conflict of Interest

The authors declare that the research was conducted in the absence of any commercial or financial relationships that could be construed as a potential conflict of interest.
