# Supplementary material for: Subarachnoid Hemorrhage Increases Level of Heme Oxygenase-1 and Biliverdin Reductase in the Choroid Plexus
Source: Front Cell Neurosci. 2020 Nov 26;14:593305. doi: 10.3389/fncel.2020.593305 (PMC7732689; doi:10.3389/fncel.2020.593305)
Supplement: Supplementary file 1 [file Data_Sheet_1.PDF]

frontiers

Supplementary Material

Supplementary Data

Supplementary material 1: Western blot analyses of HO-1 and BVR in CP. The tissue samples were homogenized in PBS containing 0.1% Triton X-100 and protease and centrifuged at 10,000 g for 5 minutes at 4°C. Proteins were separated by SDS-polyacrylamide gel electrophoresis and transferred to nitrocellulose membranes by electroblotting. Primary antibodies: anti HO-1 rabbit polyclonal antibody (1:250; ab13242, Abcam; USA) or anti BVR rabbit polyclonal antibody (1:250; PA1-26089, Thermo Fisher Scientific; USA). Secondary antibody: peroxidase-conjugated anti-rabbit IgG (Sigma, 1:1000). Protein bands were visualized using the ECL detection kit.

Supplementary Figures

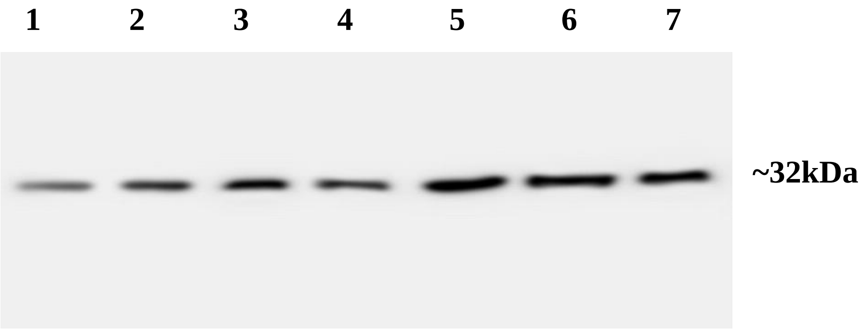

Supplementary Figure 1. Western blot of HO-1 in the CP from naïve, ACSF, and SAH rats at 1, 3 and 7 days after the operation. Legend: 1. Naive, 2. ACSF 1D, 3. ACSF 3D. 4 ACSF 7D, 5. SAH 1D, 6. SAH 3D, 7. SAH 7D.

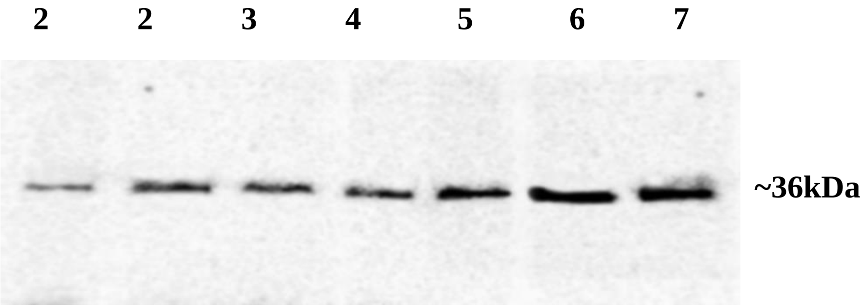

Supplementary Figure 2. Western blot of BVR in the CP from naïve, ACSF, and SAH rats at 1, 3, and 7 days after the operation. Legend: 1. Naive, 2. ACSF 1D, 3. ACSF 3D. 4 ACSF 7D, 5. SAH 1D, 6. SAH 3D, 7. SAH 7D.

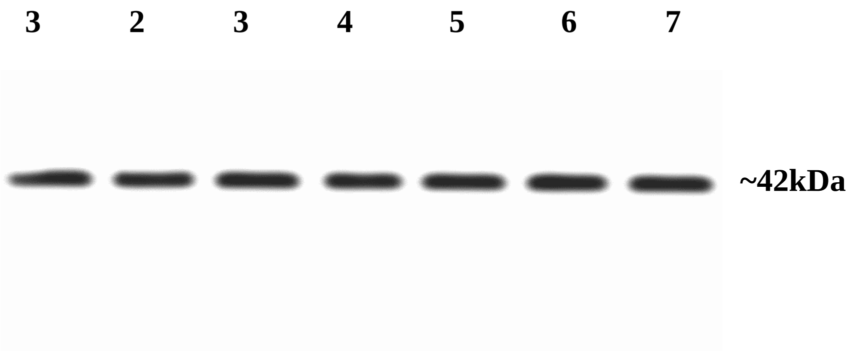

Supplementary Figure 3. Western blot of actin in the CP from naïve, ACSF, and SAH rats at 1, 3, and 7 days after the operation. Legend: 1. Naive, 2. ACSF 1D, 3. ACSF 3D. 4 ACSF 7D, 5. SAH 1D, 6. SAH 3D, 7. SAH 7D.
